# Hepatic arterial infusion chemotherapy combined with immune checkpoint inhibitors and molecular targeted therapies for advanced infiltrative hepatocellular carcinoma: a single-center experience

**DOI:** 10.3389/fimmu.2024.1474442

**Published:** 2025-01-10

**Authors:** Zizhuo Wang, Songlin Song, Lijie Zhang, Tingting Yang, Wei Yao, Bin Liang

**Affiliations:** ^1^ Department of Radiology, Hubei Key Laboratory of Molecular Imaging, Union Hospital, Tongji Medical College, Huazhong University of Science and Technology, Wuhan, China; ^2^ Hubei Provincial Clinical Research Center for Precision Radiology & Interventional Medicine, Wuhan, China; ^3^ Hubei Province Key Laboratory of Molecular Imaging, Wuhan, China

**Keywords:** infiltrative hepatocellular carcinoma, hepatocellular carcinoma, hepatic arterial infusion, immunotherapy, molecular targeted therapy

## Abstract

**Background:**

Infiltrative hepatocellular carcinoma (HCC) remains a therapeutic challenge due to its aggressive course and poor prognosis. Hepatic arterial infusion chemotherapy (HAIC) plus immune checkpoint inhibitors (ICIs) and molecular targeted therapies (MTTs) has shown early promise for advanced HCC, but its role in advanced infiltrative HCC is unclear. This study aims to investigate the efficacy and safety of HAIC combined with ICIs and MTTs in the treatment of advanced infiltrative HCC.

**Methods:**

Patients with infiltrative HCC initially treated with HAIC plus ICIs and MTTs were consecutively included at our institution from November 2021 to June 2023. The efficacy evaluation included tumor response, time to response (TTR), duration of response (DOR), progression-free survival (PFS) per RECIST 1.1, and patient survival. Adverse events (AEs) were recorded for safety evaluation.

**Results:**

A total of 27 patients were included and the median follow-up was 15.8 months (range, 4.3–25.9). The best objective response rate (ORR) and disease control rate (DCR) were 70.4% and 88.9%, respectively. The median TTR was 2.8 months (95% confidence interval [CI], 2.6–3.0) and the median DOR was 7.9 months (95% CI, 3.2–12.5). The median PFS was 7.5 months (95% CI, 4.2–10.7), and the median overall survival (OS) was 16.8 months (95% CI, 14.0–19.6), with a 1-year OS rate of 74.1%. No cases of grade 4 or 5 treatment-related adverse events (TRAEs) were observed in this study. Grade 3 TRAEs occurred in 17/27 (63.0%) patients, and the predominant grade 3 treatment-related adverse events were lymphocyte count decreased (18.5%) and neutrophil count decreased (14.8%).

**Conclusions:**

The combination of HAIC plus ICIs and MTTs demonstrated encouraging outcomes and manageable safety concerns for infiltrative HCC.

## 1 Introduction

Hepatocellular carcinoma (HCC) is the most prevalent primary liver cancer and the third most common cause of cancer-related death globally (1). HCC is highly prevalent in China, representing half of all new cases and deaths worldwide (2). Over half of these cases are diagnosed at an advanced stage (3), with a five-year survival rate estimated at just 12.1% (2). HCC can be morphologically classified into three subtypes: nodular, massive, and infiltrative (4). Infiltrative HCC is relatively rare, accounting for 7%–20% of HCC cases (4). Diagnosing infiltrative HCC is challenging because it closely resembles cirrhotic nodules, lacking distinct nodular formations and often being associated with cirrhosis. Radiologically, it manifests as tumor nodules spreading throughout the hepatic lobe or the entire liver, with unclear boundaries. Interestingly, though, cut surface samples of its small tumor nodules often reveal clear borders (4).

Most patients with infiltrative HCC are initially diagnosed at an advanced stage, presenting with macrovascular invasion and/or extrahepatic metastasis (4, 5). As a result, they are generally not candidates for curative treatments like surgical resection, liver transplantation, or ablation (4, 6), leading to a poor prognosis. Additionally, the prognosis for infiltrative HCC is worse compared to other subtypes (7), with poorer survival linked to compromised liver function (e.g., Child-Pugh score, Model for End-Stage Liver Disease score, and albumin-bilirubin grade) and higher tumor burden (e.g., elevated alpha-fetoprotein levels, vascular invasion, and extensive tumor size, number, or distribution) (5–9). Due to its aggressive nature and poor prognosis, the Barcelona Clinical Liver Cancer Staging (BCLC) system recommended systemic therapy for infiltrative intermediate stage HCC in 2022 (10).

Hepatic arterial infusion chemotherapy (HAIC) has been recommended as a first-line option for advanced HCC in Asia (3). However, local monotherapy for infiltrative HCC has been analyzed in previous studies, with HAIC reporting an objective response rate (ORR) of 34.8% and overall survival (OS) of 13.3 months (5, 6, 8, 9, 11). This highlights the urgent need for more effective treatments. In recent years, combining HAIC with molecular targeted therapies (MTTs) and immune checkpoint inhibitors (ICIs) has shown promise in advanced HCC. HAIC reduces intrahepatic tumor burden while stimulating tumor immunogen exposure to promote immunotherapy (12). When anti-angiogenic drugs are used in combination with programmed death-(ligand)1 (PD-[L]1) inhibitor, immune checkpoint activity is suppressed, and T-cell function is enhanced, leading to a stronger anti-tumor response (13, 14). Thus, this combination may have potential synergistic anti-tumor effects (15). Some real-world studies have shown that HAIC plus ICIs and MTTs for advanced HCC demonstrate a higher tumor response rate and better long-term efficacy, with an ORR of 40.0%–96.0% and a median OS of 15.9–17.9 months in the triple therapy group (16–21). We speculated that patients with the infiltrative subtype of advanced HCC could benefit from the strong anti-tumor effects of combination therapies. However, this specific approach has not yet been studied for this subtype. Therefore, this retrospective study aims to describe the efficacy and safety of HAIC with a modified FOLFOX6 regimen combined with ICIs and MTTs for antitumor treatment-naive advanced infiltrative HCC, the most malignant subtype of HCC. This study seeks to fill a gap in the literature and offer clinical insights that could shape future treatment strategies for this challenging subtype.

## 2 Materials and methods

### 2.1 Patients

This retrospective study was approved by the Ethics Committee of Union Hospital, Tongji Medical College, Huazhong University of Science and Technology (No. 2024-0725). The requirement for informed consent was waived for this retrospective study. The medical records of consecutive HCC patients who received FOLFOX-HAIC combined with ICIs and MTTs were reviewed in our institution from November 2021 to June 2023. The diagnosis and selection of the infiltrative subtype relied on imaging features, such as infiltrative or diffuse intrahepatic nodules, minimal and inconsistent arterial phase enhancement, heterogeneous washout, and no obvious boundaries in any part of the tumor on preoperative images (4). There were no restrictions on the specific use of ICIs and MTTs among the included patients, ensuring an increased sample size and representativeness.

The inclusion criteria were: 1) age 18 or older; 2) diagnosed with HCC histologically or clinically according to the European Association for the Study of the Liver (EASL) guidelines (22); 3) confirmed as infiltrative-type via CT or MRI (4); 4) Barcelona Clinical Liver Cancer (BCLC) stage C; 5) Eastern Cooperative Oncology Group (ECOG) performance status 0–1; 6) treated with a combination of HAIC with ICIs plus MTTs as the first-line treatment for at least 2 cycles. Patients were excluded if they received any other tumor-related treatment, such as transarterial chemoembolization (TACE) or radiation, during the combination therapy cycle; had other malignant tumors; had incomplete or missing clinical or imaging data; or were lost to follow-up for more than 3 months.

### 2.2 HAIC

The Seldinger technique was used to puncture the femoral artery. A 5 French catheter was inserted to identify the anatomy of the hepatic artery and the arterial supply of the tumor. A 2.7 French coaxial microcatheter was selectively placed in the feeding artery of the tumor, and perfusion chemotherapy was performed. Besides, collateral vessels should also be pre-embolized to achieve blood flow redistribution or maximize tumor control. When blood flows into the gastroduodenal artery and extrahepatic branches far from the proper hepatic artery, these routes were embolized with coils. The therapeutic scheme was a modified FOLFOX6 regimen, including oxaliplatin (85 mg/m^2^, from hour 0 to 2 on day 1), leucovorin (200 mg/m^2^, from hour 2 to 3 on day 1), and fluorouracil (400 mg/m^2^, bolus at hour 3; and 2400 mg/m^2^ over 46h on days 1 and 2). HAIC was repeated every 3 weeks until tumor progression or unacceptable toxicity. Dose reductions based on liver function and drug tolerance were permitted according to previous studies (23, 24). All patients with hepatitis B virus (HBV) infection underwent viral load testing before treatment, and received effective antiviral treatments if required.

### 2.3 Systemic treatment

Patients received intravenous PD-(L)1 inhibitors for 30–60 min every 3 weeks, with dosages as follows: sintilimab 200 mg, raltilizumab 200 mg, camrelizumab 200–250 mg, atezolizumab 1200 mg, and triplimab 240 mg. The administration of ICIs commenced on day 3 of the first HAIC cycle until disease progression or unacceptable toxicities. Dose reduction of PD-(L)1 inhibitor was not permitted, but interruption and discontinuation due to AEs were allowed.

Anti-angiogenic agents comprise tyrosine kinase inhibitors (TKIs) and monoclonal antibodies. The former, including apatinib (250 mg, once daily), donafenib (200 mg, once daily), and lenvatinib (8 mg, once daily), was administered orally. The latter involved bevacizumab (7.5 mg/kg, intravenous infusion, every 3 weeks). If HAIC was present, the oral agents were typically paused for 3–5 days during HAIC and bevacizumab was administered 3 days after HAIC. Dose reduction, interruption, and discontinuation of MTTs due to AEs were allowed.

ICIs and MTTs could be interrupted or discontinued in case any grade ≥ 3 treatment-related adverse events (TRAEs) and grade ≥ 2 immune-related adverse events (irAEs) were observed. The administration of ICIs and MTTs, including any dose adjustments, suspensions, or discontinuations, was in accordance with local care standards and the approved product label.

### 2.4 Data collection and follow-up

Baseline characteristics, clinical data, and radiological data before every treatment session for eligible patients were retrospectively collected from medical records. For the diagnosis of liver cirrhosis, non-invasive examinations, including imaging, liver function indicators, and etiology, were performed according to the EASL (25) and the Chinese Society of Hepatology Liver Cirrhosis Guidelines (26).

Chest CT and contrast-enhanced abdominal CT or MRI were performed for tumor assessments. These assessments were conducted at baseline, every 6 weeks until week 48, and then every 12 weeks until progression or death. The imaging data were independently evaluated by two radiologists (Bin Liang and Songlin Song) with over 10 years of experience. The last follow-up of this study was in June 2024.

### 2.5 Outcome

The outcome measure was progression-free survival (PFS), defined as the interval from the date of treatment initiation to the date of progression or death from any cause, whichever is sooner. Additional outcome measures included OS, 1-year OS rate, ORR, disease control rate (DCR), time to response (TTR), duration of response (DOR), and adverse events (AEs). PFS, ORR, DCR, TTR, and DOR were all assessed per RECIST 1.1. mRECIST was discarded because of atypical enhancement of infiltrative HCC. Subgroup analysis for ORR was carried out to determine the association between tumor response and the baseline characteristics. OS was defined as the interval from the date of treatment initiation to the date of death from any cause. ORR was defined as the proportion of patients with a complete response (CR) or partial response (PR). DCR was defined as the proportion of patients with a CR, PR, or stable disease (SD). TTR was defined as the time from treatment initiation to the first recorded CR or PR for patients with CR or PR. DOR was defined as the time from the first recorded CR or PR to disease progression or death for patients with CR or PR. Patients who remained alive without disease progression at the time of analysis were regarded as censored at the last imaging evaluation. TRAEs were assessed according to the National Cancer Institute Common Terminology Criteria for Adverse Events (CTCAE) version 5.0 (27) and irAEs were assessed according to the European Society for Medical Oncology (ESMO) Clinical Practice Guideline (CPG) (28).

### 2.6 Statistical analysis

Categorical variables were described as frequencies and percentages. Continuous variables were expressed as median and range (min-max). The PFS, OS, TTR, and DOR with associated 95% confidence interval (CI) were estimated using the Kaplan–Meier method. The ORR with 95% CI was determined for each subgroup, and proportion of responders between groups was compared by Fisher exact test. A two-tailed *p*-value less than 0.05 was considered to indicate statistically significant differences. Statistical analysis was performed using SPSS 27.0 (SPSS, Chicago, IL, USA) and GraphPad Prism 10.0 (GraphPad Software, La Jolla, CA, USA).

## 3 Result

### 3.1 Patient characteristics

**Figure 1:**
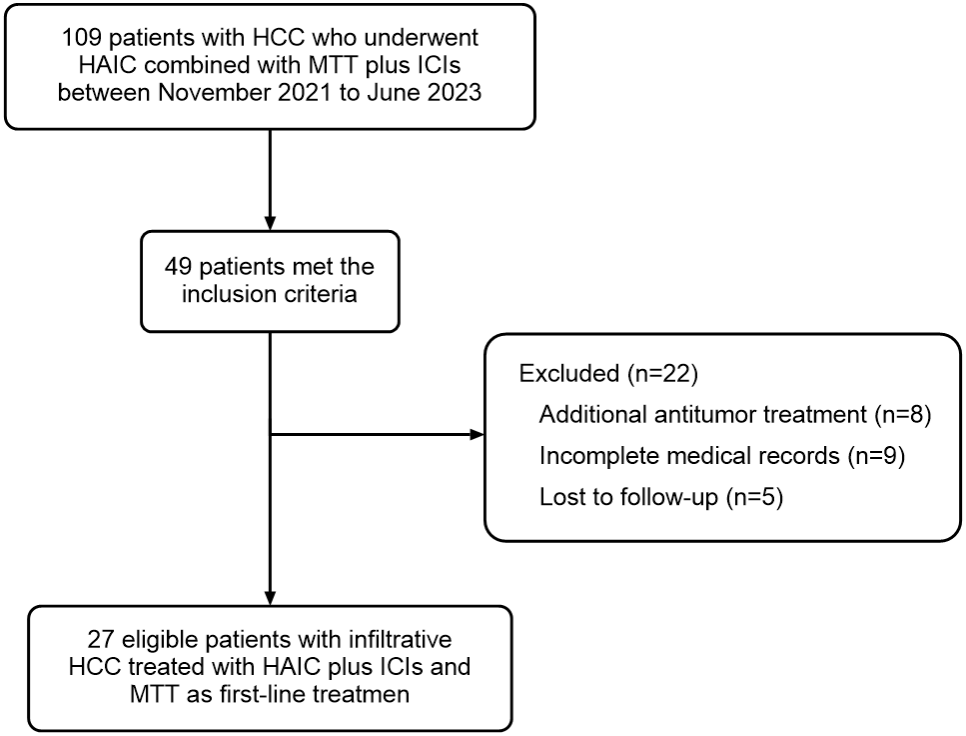
Patient flowchart. A total of 109 patients were screened, of which 49 cases met the inclusion criteria. Twenty-two of these 49 cases met the exclusion criteria: 8 cases received additional antitumor treatment, 9 cases had incomplete medical records, and 5 cases were lost to follow-up. Finally, 27 patients were included in this study and analyzed for efficacy and safety.

A total of 49 infiltrative HCC patients initially received HAIC combined with ICIs plus MTTs in our hospital between November 2021 and June 2023. Of those, 8 were excluded because they received other tumor-related treatment in addition to the combination therapy, 9 were excluded because of incomplete medical records, and 5 were lost to follow-up. Finally, 27 patients were included in this study (**Figure 1**). The median age was 54 years (range, 29–78). The median tumor diameter was 13.0 cm (range, 3.4–23.8), and 21 patients (77.8%) had tumor size ≥10 cm. The most prevalent cause of HCC was chronic hepatitis B virus infection (88.9%), and 77.8% of patients had cirrhosis. More than half of patients (63.0%) were classified as Child-Pugh class A liver function. All 27 included patients had BCLC stage C disease, of which 24 (88.9%) had portal invasion, and 12 (44.4%) had extrahepatic spread. The baseline characteristics are summarized in **Table 1**.

**Table 1:** Baseline characteristics of patients.

Characteristics	Patients (n = 27)
Sex, No. (%)	
Male	24 (88.9)
Female	3 (11.1)
Age, meidan (range), y	54 (29–78)
Age, No. (%)	
<55	14 (51.9)
≥55	13 (48.1)
Diagnosis method, No. (%)	
Histological	16 (59.3)
Clinical	11 (40.7)
Comorbidities, No. (%)	11 (40.7)
Hypertension	6 (22.2)
Diabetes	3 (11.1)
Heart disease	2 (7.4)
Others	7 (25.9)
Etiology, No. (%)	
Hepatitis B	24 (88.9)
Hepatitis C	3 (11.1)
Cirrhosis, No. (%)	
Absent	6 (22.2)
Present	21 (77.8)
Child-Pugh class, No. (%)	
A	17 (63.0)
B	10 (37.0)
ALBI grade, No. (%)	
1	7 (25.9)
2	18 (66.7)
3	2 (7.4)
ECOG PS, No. (%)	
0	14 (51.9)
1	13 (48.1)
Portal invasion, No. (%)	24 (88.9)
Vp 0	3 (11.1)
Vp 1-2	5 (18.5)
Vp 3	9 (33.3)
Vp 4	10 (37.0)
IVCTT, No. (%)	
Absent	21 (77.8)
Present	6 (22.2)
Extrahepatic spread, No. (%)	
Absent	15 (55.6)
Present	12 (44.4)
Lung	4 (14.8)
Lymph nodes	9 (33.3)
Other	3 (11.1)
Multiple organ	3 (11.1)
Tumor distribution, No. (%)	
Unilobar	10 (37.0)
Bilobar	17 (63.0)
Tumour number, No. (%)	
≤3	1 (3.7)
>3	26 (96.3)
Tumour size, meidan (range), cm	13.0 (3.4–23.8)
Tumour size, No. (%)	
<10	6 (22.2)
≥10	21 (77.8)
AFP, No. (%), ng/mL	
<400	9 (33.3)
≥400	18 (66.7)

ALBI, albumin-bilirubin grade; ECOG PS, Eastern Cooperative Oncology Group Performance Status; IVCTT, inferior vena cava tumor thrombosis; AFP, α-fetoprotein.

### 3.2 Efficacy

The median follow-up was 15.8 months (range, 4.3–25.9). The administration of study treatment is shown in **Table 2**. The median number of HAIC cycles was 6 (range, 3–9); the median number of ICIs cycles was 7 (range, 3–18), and the median duration for MTTs was 7.0 months (range, 3.0–17.0). The most commonly used anti-PD-(L)1 agent and targeted drug were sintilimab (44.4%) and bevacizumab (44.4%), respectively. At the time of analysis, 20 (74.1%) patients had met the primary endpoint; of these, 15 patients (55.6%) had disease progression, and five patients (18.5%) had died. Those 15 patients received subsequent treatment. Subsequent treatment after discontinuation of study treatment is shown in **Table 3**.

**Table 2:** The administration of study treatment.

Study treatment	Patients (n = 27)
Treatment management, median (range)	
HAIC cycle	6 (3–9)
ICIs cycle	7 (3–18)
Duration of MTTs, month	7.0 (3.0–17.0)
PD-(L)1 inhibitor, No. (%)	
Sintilimab	12 (44.4)
Raltilizumab	6 (22.2.0)
Camrelizumab	6 (22.2)
Atezolizumab	2 (7.4)
Triplimab	1 (3.7)
Targeted drug, No. (%)	
Apatinib	2 (7.4)
Donafenib	4 (14.8)
Lenvatinib	9 (33.3)
Bevacizumab	12 (44.4)

HAIC, hepatic arterial infusion chemotherapy; ICIs, immune checkpoint inhibitors; MTTs, molecular targeted therapies; PD-(L)1, programmed death-(ligand)1.

**Table 3:** Subsequent treatment after discontinuation of study treatment.

Subsequent treatments, number	Patients (n=15)
Continuation of the original program	1
Transarterial chemoembolization	2
Other systemic chemotherapy	
Sintilimab plus lenvatinib	3
Camrelizumab plus apatinib	1
Tirelizumab plus lenvatinib	1
Atrizumab plus bevacizumab	1
Regorafenib	3
Sintilimab	1
Conservative therapy	2

**Figure 2:**
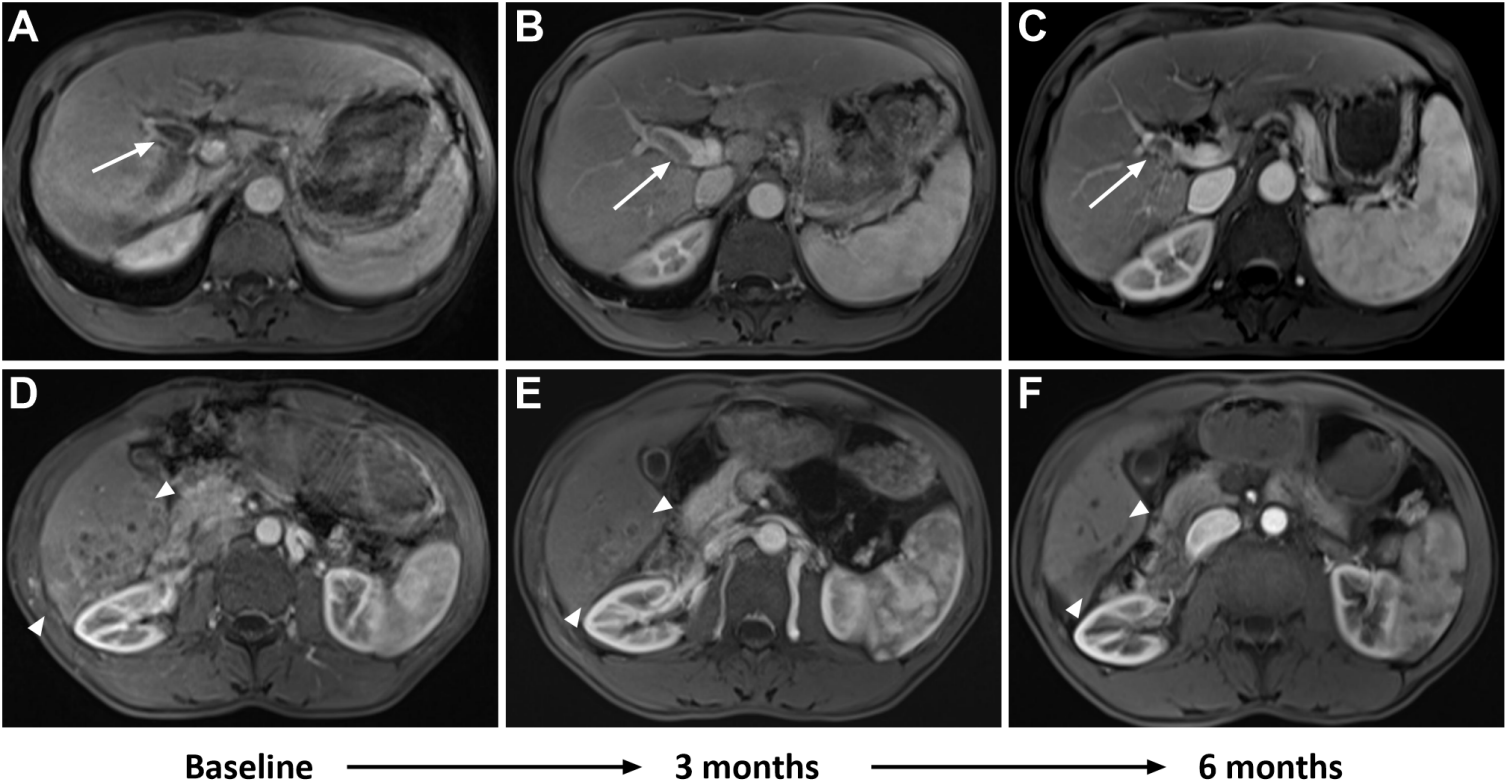
A 53-year-old man had advanced infiltrative hepatocellular carcinoma (HCC) confirmed by histology. Baseline MRI showed a right portal vein tumor thrombus (PVTT) in the portal venous phase (**A**, white arrow). Follow-up imaging after combination therapy showed a reduction in PVTT at 3 months (**B**, white arrow), with further reduction at 6 months (**C**, white arrow). Additionally, baseline MRI revealed patchy areas of heterogeneous enhancement diffusely involving the posterior segment of the right lobe in the arterial phase (**D**, between white arrowheads). Follow-up imaging after combination therapy showed a decrease in tumor size and partial response (PR) at 3 months (**E**, between white arrowheads), with further reduction and loss of enhancement observed at 6 months (**F**, between white arrowheads).

**Figure 3:**
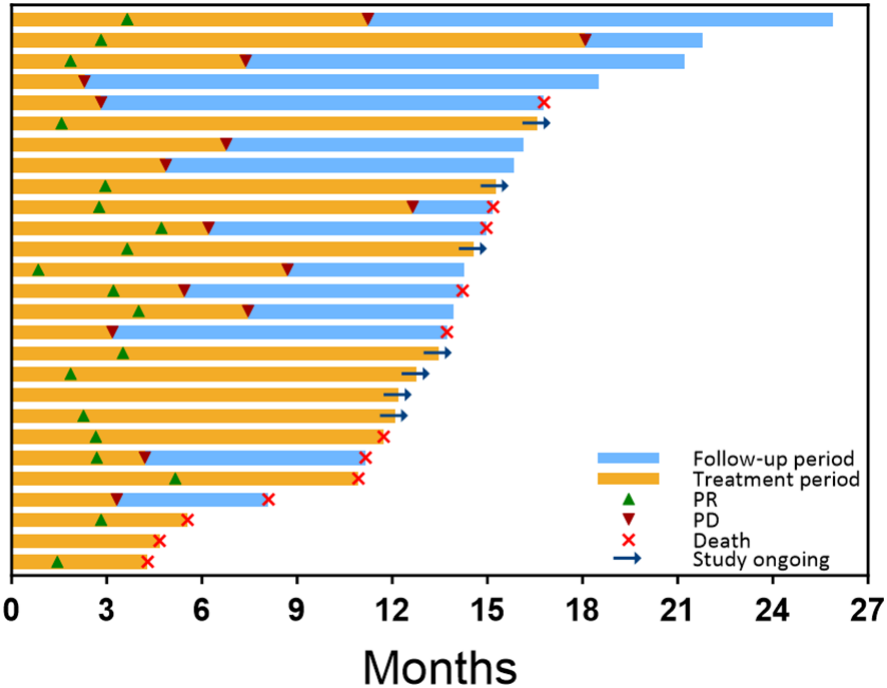
Treatment exposure, survival, and response duration for all patients, as assessed by RECIST 1.1, were ranked based on the follow-up period after the initial combination therapy. The orange bar indicates the duration of combined treatment for each patient. Green triangles mark the time of the first PR, while red triangles denote the time of progressive disease (PD) on imaging. The blue bar represents the survival follow-up period following PD. At the end of each patient’s follow-up period, a red cross signifies the time of death, while a right arrow indicates that the patient is still receiving combination therapy. Patients who are not marked with a red cross or right arrow are still under survival follow-up.

**Figure 4:**
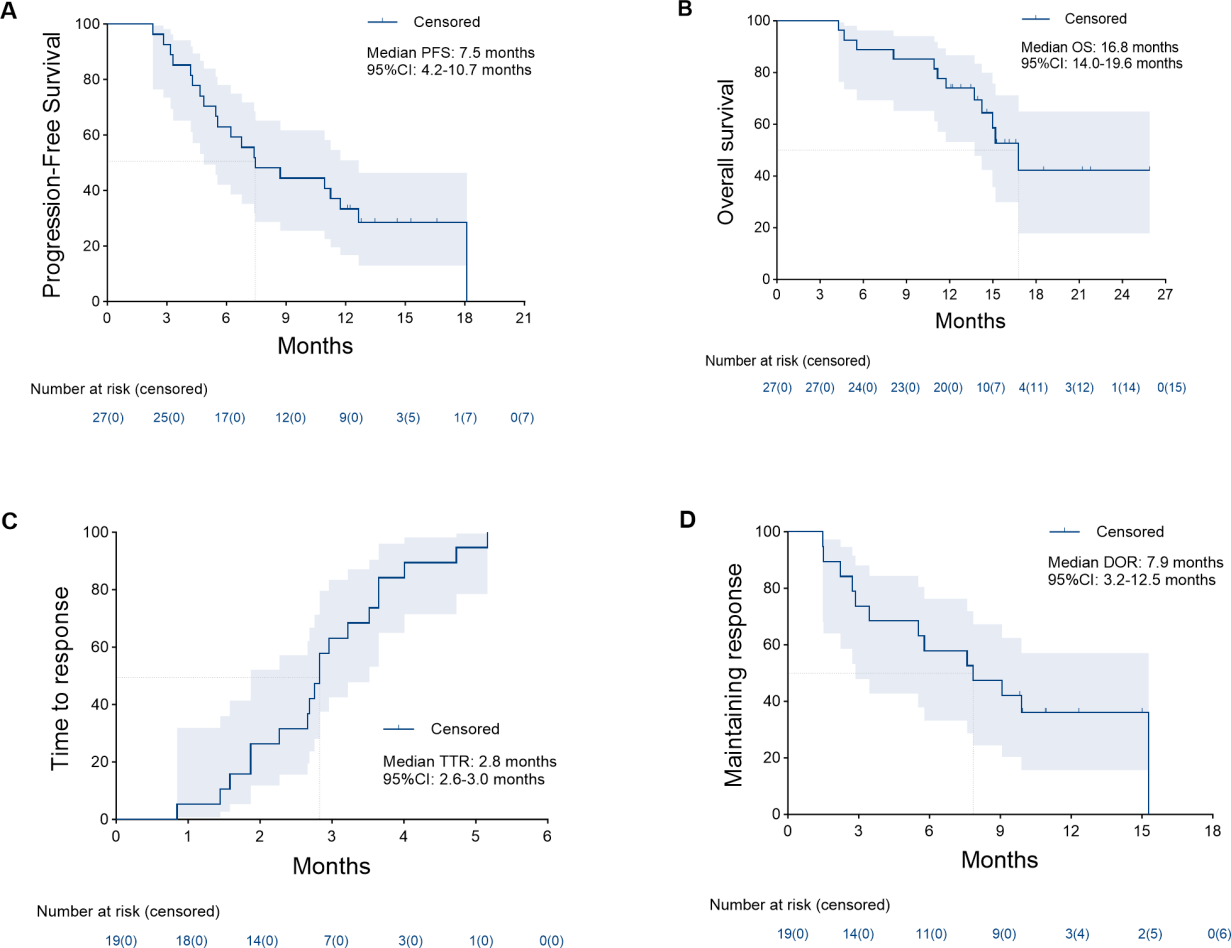
Kaplan–Meier analysis of the median progression-free survival **(A)** was 7.5 months (95% confidence interval [CI], 4.2–10.7), median overall survival **(B)** was 16.8 months (95% CI, 14.0–19.6), median time to response **(C)** was 2.8 months (95% CI, 2.6–3.0), and median duration of response **(D)** was 7.9 months (95% CI, 3.2–12.5).

Tumor response is shown in **Table 4**. At the 3-month time point, the ORR and DCR were 51.9% and 88.9%, respectively. According to the best objective response, the ORR and DCR were 70.4% and 88.9%, respectively (**Figure 2**). The median TTR and DOR were 2.8 months (95% CI, 2.6–3.0) and 7.9 months (95% CI, 3.2–12.5), respectively (**Table 5**, **Figure 3**). The median PFS was 7.5 months (95% CI, 4.2–10.7), with the 3-, 6-, and 12-month PFS rates of 92.6%, 63.0%, and 33.3%, respectively. The median OS was 16.8 months (95% CI, 14.0–19.6), with a 1-year OS rate of 74.1% (**Figure 4**). Subgroup analysis of the ORR (**Table 6**) showed that ORR was consistent in all subgroups. Although patients with ALBI grade 1, no extrahepatic metastases, and unilobar involvement demonstrated higher tumor response rates, the differences were not statistically significant.

**Table 4:** Tumor response.

Variables, No. (%)	RECIST 1.1 (n=27)	
	Time point response at 3 months	Best response
ORR	14 (51.9)	19 (70.4)
DCR	24 (88.9)	24 (88.9)
Overall response		
CR	0	0
PR	14 (51.9)	19 (70.4)
SD	10 (37.0)	5 (18.5)
PD	3 (11.1)	3 (11.1)
Intrahepatic response		
CR	0	0
PR	16 (59.3)	21 (77.8)
SD	11 (40.7)	6 (22.2)
PD	0	0

ORR, objective response rate; DCR, disease control rate; CR, complete response; PR, partial response; SD, stable disease; PD, progressive disease.

**Table 5:** Summary of Efficacy Outcomes.

Variables	RECIST 1.1 (n=27)
PFS, median (95% CI), month	7.5 (4.2–10.7)
Patients with events, No. (%)	20 (74.1)
PD	15 (55.6)
New lesions of lung	3 (11.1)
New lesions of bone	1 (3.7)
New lesions of abdominal cavity	1 (3.7)
New lesions of liver	7 (25.9)
Progression of intrapulmonary lesions	2 (7.4)
Progression of intrahepatic lesions	1 (3.7)
Death	5 (18.5)
PFS rate, %	
3m	92.6
6m	63.0
12m	33.3
TTR, median (95% CI), month	2.8 (2.6–3.0)
DOR, median (95% CI), month	7.9 (3.2–12.5)
OS, median (95% CI), month	16.8 (14.0–19.6)
1-year OS rate, %	74.1

CI, confidence interval; PFS, progression-free survival; TTR, time to response; DOR, duration of response; OS, overall survival.

**Table 6:** Subgroup analysis of objective response rate.

Groups	n/N	ORR (95% CI)	*P-*value
All patients	19/27	70.4% (51.9–88.9)	–
Sex			0.532
Male	16/24	66.7% (45.8–83.3)	
Female	3/3	100.0% (100.0–100.0)	
Age			0.678
≥55	10/13	76.9% (53.8–100.0)	
<55	9/14	64.3% (35.7–85.7)	
Comorbidities			1.000
Present	8/11	72.7% (45.5–100.0)	
Absent	11/16	68.8% (43.8–87.5)	
Cirrhosis			1.000
Present	15/21	71.4% (52.4–90.5)	
Absent	4/6	66.7% (17.1–100.0)	
Child-Pugh class			1.000
B	7/10	70.0% (40.0–100.0)	
A	12/17	70.6% (47.1–88.2)	
ALBI grade			0.633
2-3	13/20	65.0% (45.0–85.0)	
1	6/7	85.7% (57.1–100.0)	
ECOG PS			0.420
1	8/13	61.5% (38.5–84.6)	
0	11/14	78.6% (57.1–100.0)	
Portal invasion			1.000
Vp 3-4	13/19	68.4% (47.4–89.5)	
Vp 0-2	6/8	75.0% (37.8–100.0)	
IVCTT			1.000
Present	4/6	66.7% (33.3–100.0)	
Absent	15/21	71.4% (52.4–90.5)	
Extrahepatic spread			0.398
Present	7/12	58.3% (33.3–83.3)	
Absent	12/15	80.0% (60.0–100.0)	
Tumor distribution			0.190
Unilobar	10/17	58.8% (35.3–82.4)	
Bilobar	9/10	90.0% (70.0–100.0)	
Tumour size			1.000
≥10 cm	15/21	71.4% (52.4–90.5)	
<10 cm	4/6	66.7% (33.3–100.0)	
AFP level			1.000
≥400 ng/mL	13/18	72.2% (50.0–88.9)	
<400 ng/mL	6/9	66.7% (33.3–100.0)	

ALBI, albumin-bilirubin grade; ECOG PS, Eastern Cooperative Oncology Group Performance Status; IVCTT, inferior vena cava tumor thrombosis; AFP, α-fetoprotein; CI, confidence interval.

### 3.3 Safety

TRAEs of grade 4 and 5 did not occur in this study. All patients experienced at least one TRAE, and 17 patients (63.0%) experienced at least one grade 3 AE (**Table 7**). The common TRAEs of any grade included hypoalbuminemia (59.3%) and lymphocyte count decreased (48.1%). The predominant grade 3 AEs were lymphocyte count decreased (18.5%) and neutrophil count decreased (14.8%). For patients experiencing grade 3 gastrointestinal hemorrhage (7.4%), the combination therapy was interrupted. Endoscopic treatment for bleeding was administered, and the combination therapy was resumed only after recovery to grade 0–1, in conjunction with gastric mucosal protective medications. Furthermore, a particular abdominal pain associated with oxaliplatin was observed in 7 patients. This pain was quickly relieved by slowing or stopping the infusion of oxaliplatin though it could be acute and severe.

**Table 7:** Treatment-related adverse events.

Events, No. (%)	All patients (n = 27)		
	Any grade	Grades 1-2	Grade 3
Any treatment-related adverse event^*^	27 (100.0)	27 (100.0)	17 (63.0)
Hypoalbuminemia	16 (59.3)	14 (51.9)	2 (7.4)
Lymphocyte count decreased	13 (48.1)	8 (29.6)	5 (18.5)
Thrombocytopenia	11 (40.7)	8 (29.6)	3 (11.1)
Neutrophil count decreased	11 (40.7)	7 (25.9)	4 (14.8)
Aspartate aminotransferase increased	9 (33.3)	9 (33.3)	0
Hyperbilirubinemia	9 (33.3)	8 (29.6)	1 (3.7)
Abdominal pain	8 (29.6)	8 (29.6)	0
Hypertension	8 (29.6)	7 (25.9)	1 (3.7)
Alanine aminotransferase increased	7 (25.9)	6 (22.2)	1 (3.7)
Nausea	7 (25.9)	7 (25.9)	0
Anemia	7 (25.9)	6 (22.2)	1 (3.7)
Proteinuria	6 (22.2)	6 (22.2)	0
Gastrointestinal hemorrhage	6 (22.2)	4 (14.8)	2 (7.4)
Diarrhea	4 (14.8)	3 (11.1)	1 (3.7)
Anorexia	2 (7.4)	2 (7.4)	0
Weight loss	2 (7.4)	2 (7.4)	0
Ascites/pleural effusion	2 (7.4)	1 (3.7)	1 (3.7)
Hypothyroidism	1 (3.7)	1 (3.7)	0
Handefoot skin reaction	1 (3.7)	1 (3.7)	0
Immune-related adverse event^#^	9 (33.3)	7 (25.9)	2 (7.4)
Immune-related dermatitis	3 (11.1)	2 (7.4)	1 (3.7)
Immune-related enterocolitis	2 (7.4)	1 (3.7)	1 (3.7)
Immune-related hypothyroidism	2 (7.4)	2 (7.4)	0
Immune-related hepatitis	1 (3.7)	1 (3.7)	0
Immune-related pneumonitis	1 (3.7)	1 (3.7)	0

^*^ Treatment-related adverse events were assessed according to the National Cancer Institute Common Terminology Criteria for Adverse Events (CTCAE) version 5.0.

^#^ Immune-related adverse events were assessed according to the European Society for Medical Oncology (ESMO) Clinical Practice Guideline (CPG).

A total of 9 patients (33.3%) experienced irAEs. The predominant irAEs of any grade were dermatitis (11.1%), enterocolitis (7.4%), and hypothyroidism (7.4%). irAEs of grade 3 were evident in 2 patients (7.4%), with the predominant events being dermatitis (3.7%), characterized by erythematous papules on the limbs and severe pruritus, and enterocolitis (3.7%), manifested by severe diarrhea (increase of ≥7 stools/day). These conditions caused discontinuation and interruption of immunotherapy, respectively, and both recovered after steroid treatment.

AEs prompted dose discontinuation and interruption of PD-(L)1 inhibitor occurred in 1/27 (3.7%) and 3/27 (11.1%) patients, respectively. AEs prompted dose discontinuation, interruption, and reduction of targeted drugs occurred in 2/27 (7.4%), 3/27 (11.1%), and 4/27 (14.8%) patients, respectively. No patients discontinued HAIC treatment due to AEs. AEs leading to dose adjustments are summarized in **Table 7**.

## 4 Discussion

The combination of HAIC with ICIs and MTTs showed promise in improving outcomes in patients with infiltrative HCC. This retrospective study, to the best of our knowledge, is the first study to evaluate the efficacy and safety of HAIC combined with ICIs and MTTs in patients with BCLC stage C infiltrative HCC who had not previously undergone any form of antitumor treatment. In this research, HAIC plus PD-(L)1 inhibitors and anti-angiogenic agents in patients with antitumor treatment-naive infiltrative HCC yielded a median PFS of 7.5 months, an ORR of 70.4%, and durable responses (7.9 months). The median OS was 16.8 months, with a 1-year OS rate of 74.1%. HAIC combined with ICIs plus MTTs demonstrated manageable toxicity profile. No cases of TRAEs of grade 4 or 5 were observed in this study, and grade 3 AEs occurred in 17/27 (63.0%) patients.

Our study found that HAIC combined with ICIs and MTTs in BCLC stage C infiltrative HCC was associated with better efficacy. Previous studies have evaluated the efficacy of intra-arterial therapy in patients with infiltrative HCC, which was lower than our result in most cases. Han et al. (6) reported a median OS of 5.7 months with TACE. Kim et al. (5) found that the median PFS and OS after TACE in BCLC stage B infiltrative HCC achieved 6 months (95% CI, 5–7) and 16 months (95% CI, 14–18), respectively. An et al. (9) reported an improvement in PFS (7.8 *vs* 4.0 months, *P* = 0.035) and OS (13.3 *vs* 10.8 months, *P* = 0.043) with HAIC over TACE in infiltrative HCC patients with Child-Pugh class A. The PFS in the HAIC group, as observed in the study by An et al., was consistent with our findings, and the OS in our study was longer. However, this study included more patients with comorbidities (40.7% *vs* 13.2%), a larger tumor burden (13.0 cm *vs* 10.9 cm), a higher proportion of patients with vascular invasion (88.9% *vs* 70.6%), and included patients with Child-Pugh B (37.0%). The prognosis of these patients is often considered poor (5, 29–31) and often excluded from most other studies for advanced HCC (19, 32, 33). Therefore, the population included in our study may have better reflected the population typically observed in routine clinical practice for advanced HCC.

Although the results of this study showed advances in the treatment of infiltrative HCC, the curative effect was unsatisfactory. A high tumor burden is recognized as an independent prognostic factor for HCC, with a stronger tumor response being associated with improved survival outcomes (8, 9). In this study, the ORR and DCR were 70.4% and 88.9%, respectively. Given the aggressive progression characteristic of infiltrative HCC, the time point response at 3 months was selected to assess ORR and DCR. At this time point, the ORR was 51.9%, and the DCR remained at 88.9%, with a TTR of 2.8 months (95% CI, 2.6–3.0). Prior studies reported an ORR of 10.7% to 12.1% for advanced infiltrative HCC following TACE (6, 9, 34), while 34.8% after HAIC (9). When compared to the results from studies on triple combination therapy for advanced HCC (18, 19, 31, 32), our findings present a contrasting picture. While the ORR in previous studies was comparable (77.1%) or even lower (54.1% to 63.9%) than in our study, these studies reported longer PFS of 10.4 to 11.1 months and OS of 17.9 months or not reached. These results may align with the dismal prognosis associated with the infiltrative subtype of HCC. Although infiltrative HCC shows a rapid and favorable tumor response with triple therapy, the malignancy appears to remain aggressive, with a high likelihood of relapse or progression following an early ORR. Our study sought to determine whether tumor response was associated with different subgroups within the patient population. Unfortunately, the tumor response of the triple therapy was consistent across different patient subgroups, even though patients with ALBI grade 1, absence of extrahepatic metastases, and unilobar involvement showed higher tumor responses. This may be attributed to the rarity of the subtype and the limited sample size.

Triple therapy has shown potential benefits in advanced HCC and is also expected to enhance outcomes in the advanced infiltrative subtype. The possible main reason for the significant anti-tumor activity might be the synergistic impact of the triple combination treatment with HAIC, PD-(L)1 inhibitors, and targeted drugs. Firstly, HAIC can enhance the local hepatic drug concentration and the penetration of drugs into tumors, maximizing the effectiveness of chemotherapy while reducing systemic toxicity (35). Secondly, targeted drugs and PD-(L)1 inhibitors play a crucial role in modulating the tumor immune microenvironment and enhancing immune response (19, 33, 36, 37). This combination may potentially reduce resistance to anti-angiogenic drugs by disrupting an immunosuppressive environment (38). Thirdly, the synergy between targeted drugs and PD-(L)1 inhibitors may impede tumor angiogenesis, promote vascular normalization, and overcome resistance to FOLFOX agents (31, 39, 40), thereby disrupting tumor hypoxia and enhancing drug delivery and permeation. Lastly, the immunogenic cell death induced by HAIC further augments the anti-tumor effect of immunotherapy (41, 42). Both targeted drugs and the sustained high-concentration chemotherapy of HAIC have demonstrated efficacy in managing patients with a high tumor burden and effectively improving tumor regression rates (18, 19, 23, 43, 44). Therefore, triple therapy holds the potential to promptly alleviate tumor burden and prolong the duration of the systemic treatment response.

In this study, no treatment-related deaths occurred, and the safety profile was similar to that of previous combination therapies for advanced HCC (18, 19, 31, 32). There were no unexpected adverse events observed. Two patients experienced grade 3 gastrointestinal hemorrhage, which was attributed to bevacizumab, and both recovered by promptly discontinuing the combination treatment and receiving urgent treatment. Therefore, establishing strict inclusion criteria for patients at high risk of bleeding is of paramount importance. Additionally, although grade 3 lymphocyte count decreased (18.5%) and neutrophil count decreased (14.8%) were common, these side effects were associated with HAIC and improved quickly in the short term, without significantly impacting treatment. An increase in irAEs of any grade was observed, however, the majority of cases were grades 1–2 and were manageable. No other potential toxic events were observed, demonstrating that the combination therapy for infiltrative HCC is feasible and safe.

### 4.1 Limitations of the study

There are several limitations of our study that need to be mentioned and further discussed. First, he mRECIST criteria were not used to evaluate efficacy in this study due to the challenges it presents in assessing tumor response in infiltrative HCC. In infiltrative HCC, arterial enhancement is typically minimal and inconsistent, and the appearance of washout in the portal venous phase is often irregular and heterogeneous, which does not align with the typical enhancement characteristics of HCC. This inconsistency fails to meet mRECIST requirements for identifying intrahepatic target lesions (viable tumors) with classic HCC enhancement features, such as greater enhancement than the surrounding liver parenchyma during the arterial phase and a washout appearance during the portal venous phase. In contrast, RECIST 1.1 defines lesion measurement by the longest overall tumor diameter, irrespective of enhancement or internal necrotic areas (45). Thus, RECIST 1.1 appears more suitable than mRECIST for evaluating infiltrative HCC. Further studies are needed to explore methods for evaluating tumor activity in infiltrative HCC. Second, this study was a single-center retrospective study with a relatively small sample size, so selection bias could not be completely avoided. This is primarily due to the rarity of infiltrative HCC and the stringent screening criteria used to minimize patient heterogeneity. However, these factors may limit the generalizability of our findings to patients with different disease stages, prior antitumor treatments, or from different regions. Future prospective randomized controlled trials or large multicenter studies with patients at different disease stages and treatment backgrounds are needed to validate our findings and improve the generalizability and applicability of the results in clinical settings. Third, the follow-up period was relatively short, limiting the assessment of long-term treatment effects. The highly malignant nature of infiltrative HCC, which leads to reduced survival, also contributed to the shorter follow-up. Additionally, short-term outcomes, such as tumor response and PFS, are not influenced by subsequent treatments, making them more accurate measures of efficacy than OS. Meanwhile, patients in this study are still under ongoing follow-up to obtain long-term data on the efficacy of the combination therapy.

### 4.2 Future recommendations

The influence of the infiltrative subtype on clinical trial outcomes may be noteworthy. Previous studies often lacked data on the specific tumor types of HCC or the proportion of infiltrative HCC, which could skew results, as a higher proportion of the infiltrative subtype might lead to poorer prognosis. This underscores the importance of stratifying populations based on tumor subtype to more accurately assess treatment efficacy. Additionally, infiltrative HCC has shown a high tumor response when assessed by tumor size as defined by RECIST 1.1, which might underestimate its malignancy. To more accurately reflect the poor prognosis associated with this subtype, more stringent methods for evaluating tumor response are needed, such as combining measurements of tumor size with enhancement. For example, a patient assessed as a PR based on tumor size should be classified as SD rather than PR if lesion still shows enhancement.

## 5 Conclusion

In summary, HAIC combined with PD-(L)1 inhibitors and targeted drugs appears to have a favorable anti-tumor activity in patients with advanced, treatment-naive infiltrative HCC, with manageable toxicities. Although the outcomes are still below optimal expectations, this triple therapy presents a viable and promising alternative for treating this challenging HCC subtype, which warrants further investigation.

## Data Availability

The original contributions presented in the study are included in the article/supplementary material. Further inquiries can be directed to the corresponding author.
